# Options to Improve the Mechanical Properties of Protein-Based Materials

**DOI:** 10.3390/molecules27020446

**Published:** 2022-01-10

**Authors:** Anne Lamp, Martin Kaltschmitt, Jan Dethloff

**Affiliations:** Institute of Environmental Technology and Energy Economics (IUE), Hamburg University of Technology (TUHH), Eißendorfer Straße 40, 21073 Hamburg, Germany; anne.lamp@tuhh.de (A.L.); martin.kaltschmitt@tuhh.de (M.K.)

**Keywords:** protein-based materials, home compostability, mechanical properties, cross-linking, plasticization, protein structure

## Abstract

While bio-based but chemically synthesized polymers such as polylactic acid require industrial conditions for biodegradation, protein-based materials are home compostable and show high potential for disposable products that are not collected. However, so far, such materials lack in their mechanical properties to reach the requirements for, e.g., packaging applications. Relevant measures for such a modification of protein-based materials are plasticization and cross-linking; the former increasing the elasticity and the latter the tensile strength of the polymer matrix. The assessment shows that compared to other polymers, the major bottleneck of proteins is their complex structure, which can, if developed accordingly, be used to design materials with desired functional properties. Chemicals can act as cross-linkers but require controlled reaction conditions. Physical methods such as heat curing and radiation show higher effectiveness but are not easy to control and can even damage the polymer backbone. Concerning plasticization, effectiveness and compatibility follow opposite trends due to weak interactions between the plasticizer and the protein. Internal plasticization by covalent bonding surpasses these limitations but requires further research specific for each protein. In addition, synergistic approaches, where different plasticization/cross-linking methods are combined, have shown high potential and emphasize the complexity in the design of the polymer matrix.

## 1. Introduction

Bio-based and biodegradable materials have shown strong growth in the packaging industry mainly due to recent trends in the consumer market moving toward greener packaging and reducing waste products [1]. With the increasing concern on sustainability politics, academia and industry are encouraged to develop sustainable and circular alternatives for preserving limited resources for future generations, focusing on biodegradable and bio-renewable materials [2].

In the recent past, the main focus has been put on biopolymers derived from synthetization from bio-based monomers, such as polylactic acid (PLA) or polyhydroxyalkanoates (PHA). Especially in the packaging industry, such macro-molecules are commonly considered as potential replacements to fossil-based polymers [1]. However, an EU directive introduced in July 2019 bans the single-use of non-compostable materials such as plastics by 2021 and threatens a find to any countries who do not respect these obligations. In parallel, for products intended to be disposed of after one use cycle, degradation under natural conditions must be guaranteed to assure a sustainable removal from the natural environment. To allow for such home composability, under moderate temperatures between 20 and 30 °C, a biodegradation rate of 90% in 12 months maximum should be possible without any pre-treatment [3]. However, from the current bio-based polymers derived from chemical and/or microbial synthetization, none does fulfill these demands [4]. Thus, the biodegradation of these market mature bio-based polymers requires controlled composting conditions being given typically only in the industry.

**Figure 1:**
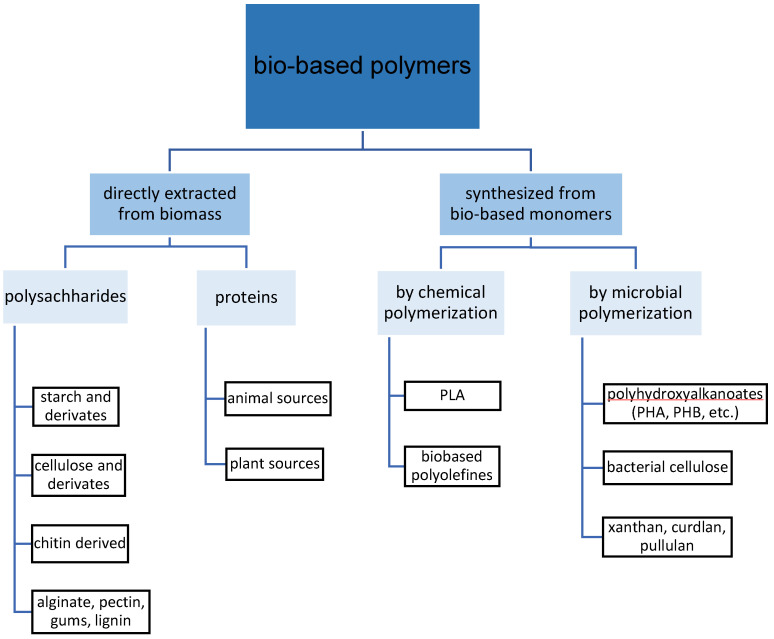
Bio-based polymers, their origin, and method of production.

Besides the synthetization from biological monomers, another option to produce bio-based materials is the use of natural polymers directly extracted from biomass (Figure 1). Natural polymers show high abundance, well-developed biocompatibility, and a clear non-toxicity [5]. Additionally, they do fulfill the criteria for microbial digestion (composability) under natural conditions [6] because they are provided by nature as polymers and can be as such returned into nature following the biological cycle of materials. From the natural polymers, polysaccharides such as cellulose or starch and their derivates show fewer promising characteristics in making hydrophobic films due to their hydrophilic structure. This results in high water interaction of materials and causes water vapor permeability, swelling, and solubilization within an aqueous environment prohibiting their usage for most material applications [7,8]. Compared to polysaccharide-based ones, protein-based materials are more useful for most material applications because of their excellent gas barrier properties and superior mechanical properties. For example, the oxygen permeability of soy protein-based films was found to be 260, 500, 540, and 670 times lower than that of low-density methylcellulose, polyethylene, starch, and pectin, respectively [9]. These advantages are due to the protein’s unique structure, which confers a wide range of functional properties, especially due to the high intermolecular binding potential [5,10,11,12,13]. However, the mechanical properties of materials produced from proteins are inferior to those of synthetic origin and do not reach the requirements for most material applications [10,14,15].

To sum up, protein-based materials show a high potential to fill the niche of home-composable materials due to their intrinsic properties but require further optimization of their mechanical stability. Within this context, this paper aims to summarize findings to improve the strength and elasticity of protein-based materials. After an introduction into the basic structural composition, the two main measures (i.e., cross-linking and plasticization) to overcome until now given limitations in the mechanical properties will be explained. Relevant case studies, ordered by the respective method, will then be presented. The influence of respective methods on other relevant material properties (e.g., the water vapor permeability) is not the primary aim of discussion. Therefore und because of the differences in protein resource, processing, and kind of manufacturing, a basis for a distinct comparison between the various methods is not given.

This review, though, serves as a collection of relevant information to scientists investigating the development of protein-based materials. It provides conclusions and a discussion part as well as a summary of all relevant data from the publications discussed here in the appendix (Table A1 and Table A2).

## 2. Protein-Based Materials

**Figure 2:**
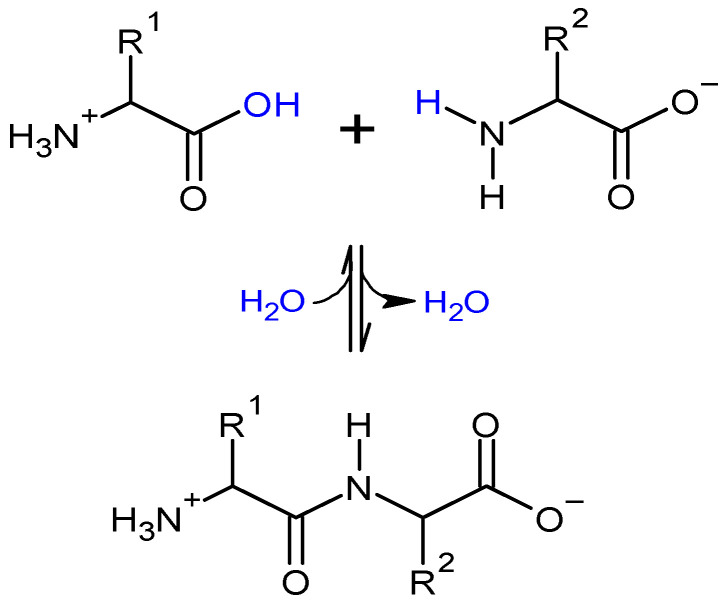
Formation of a peptide bond between two amino acids.

Proteins, generally considered to be highly complex polymers, consist of 20 different amino acids, of which are several thousand aligned to each other connected by peptide bonds (Figure 2) and forming the polymer structure. In this, the substituted amide bonds of each monomeric unit (R_i_) can be used to link the available constituents [16]. The nature of amino acid sequences and distribution and the sum of the interactions among protein chains (mainly hydrogen bonds, hydrophobic interactions, and disulphide bonds) are closely related to the mechanical properties of protein-based materials [17,18]. The promotion of polymer chain-to-chain interactions results in materials that are stronger but less flexible and also less permeable to gases, vapors, and liquids [10]. As a function of their diverse amino acid functional groups, proteins have multiple sites for chemical interaction allowing for property modifications and even tailoring [13]. Thus, the unique complexity of proteins and the diversity of their different fractions can be tapped to develop materials with optimized properties required for respective material applications.

Proteins with suitable material properties derived from animal sources include, e.g., collagen, gelatin, fish myofibrillar protein, keratin, egg white protein, casein, and whey protein. Those derived from plant sources include, e.g., corn zein, wheat gluten, soy protein, peanut protein, and horse gram protein. Even though all proteins exist of complex polymeric structures, naturally, they lack structural stability. However, over time, promising measures arose, further improving the mechanical properties of protein-based films. Plasticization of polymers is, e.g., achieved by insertion of a plasticizer into the protein matrix, disrupting existing intra- and intermolecular interactions and allocation of plasticizer molecules at the respective binding sides. Thereby, the polymer mobility and material elasticity are increased. Cross-linking of polymer chains implements the contrary effect and increases the strength of the material by the introduction of additional chain-to-chain interactions.

Applications of protein-based materials can already be found in medicine, such as tissue engineering, tablet coating, or nano-technological approaches [19,20]. This is due to their high biocompatibility and non-toxicity. Though for most packaging applications, higher structural stability to preserve the integrity of the packed goods is required. The mechanical requirements of protein-based materials can be derived from those of synthetic origin, and in general, tensile strength and elongation at break are the most relevant properties, respectively [7]. Additionally, the glass transition temperature T_g_ is a suitable indicator for plasticity because the more strongly bound the matrix components are, the greater energy (heat) is required for transition reactions to occur [5]. For a qualitative interpretation of material properties, the analysis of specific molecular interactions inside the protein matrix has been carried out by several spectroscopy techniques, Fourier transformation infrared spectroscopy (FT-IR) being the most applied one. The extent of cross-linking can be determined by either quantifying un-cross-linked ε-amino groups of the protein by UV spectrometry before and after the reaction or simply by comparing the hydrodynamic radii by SDS-PAGE or size exclusion chromatography [21].

The performance of each method certainly depends on the increase in the respective target parameter, tensile strength, and elasticity. Next to the mechanical properties, the simplicity of the technology and the degree of expense for each method shall be taken into account for assessment. Additionally, for the final product, non-toxicity must be assured, especially concerning the application of single-use items and contact with humans.

## 3. Cross-Linking

The mechanical strength and barrier properties of protein films can be improved by cross-linking methods to induce a better interaction inside and in between protein chains [15,22,23,24,25,26,27,28,29,30,31,32,33,34,35,36,37,38,39,40,41,42,43,44,45,46,47,48]. More protein interactions induced by certain methods extent the polymeric structure with the consequence that less permeability and greater tensile strength are obtained [10]. The term cross-linking describes inter- and intramolecular covalent bond formations in this context and includes disulfide bonds as well as iso-peptide bonds of any kind [18]. Cross-linking of proteins can be achieved by either chemical, physical (radiation, heat), or enzymatic treatment.

### 3.1. Chemical Cross-Linking

Chemicals used for cross-linking can interact with specific functional groups of proteins, such as the amino function in lysine or the carboxyl group in aspartic acid and glutamic acid [49]. Such cross-linking agents bind protein chains on two sides, embedding themselves in between and thus increasing chain-to-chain interactions and, therefore, the material’s strength.

#### 3.1.1. Chemical Cross-Linking with Aldehydes

Chemical agents with two aldehyde groups, particularly formaldehyde and glutaraldehyde, are widely used cross-linkers since they react quickly with free non-protonated ε-amino groups in proteins [22]. The reaction mechanism of protein cross-linking with aldehydes follows the transformation of amines into imines through the -C=N- bond between amino acids [23,50]. Although proteins intermolecular interactions are highly reactive to aldehydes, their cytotoxicity is a major disadvantage [36]. An alternative for the provision of aldehyde groups for protein cross-linking is by controlled oxidation of polysaccharides generating defined amounts of free aldehyde groups in the polysaccharide.

The use of dialdehyde alginate to cross-link and stabilize gelatin films was investigated by Boanani et al. [51]. Alginate dialdehyde was obtained through oxidation of sodium alginate, and the films were prepared from gelatin solutions at different concentrations (5, 10, and 15 wt.%) containing different amounts of oxidized alginate (0, 1, and 3 wt.% relatives to gelatin). The extent of cross-linking was monitored by UV assay of un-cross-linked ε-amino groups before and after cross-linking and increased as a function of dialdehyde alginate concentration up to about 23%. The tensile strength of the respective films could be increased from 0.7, 1.2, and 2.5 to 2.9, 2.7, and 3.4 MPa for samples with 5, 10, and 15 wt.% gelatin relative to the casting solution. In addition, a significant reduction in the degree of swelling and a small but appreciable increase in thermal stability was reported.

Also, for gelatin, the effects of different concentrations of dialdehyde starch (DAS) (5, 10, and 30 wt.% relatives to gelatin) on glycerol-plasticized films were studied by Martucci and Ruseckaite [52]. Other than for dialdehyde alginate, the tensile strength even decreased by about 30% for 5 wt.% dialdehyde starch compared to the control film.

This apparently anomalous behavior was assigned to the polymeric nature of dialdehyde starch, which does not introduce severe restrictions within the gelatin matrix. Additionally, the cross-linking effect might be counterbalanced by the plasticization exerted by the hydroxylated polymeric backbone of dialdehyde starch. Similar observations were made for the use of dialdehyde starch in feather keratin-based films [53].

#### 3.1.2. Chemical Cross-Linking with Poly-Phenols

**Figure 3:**
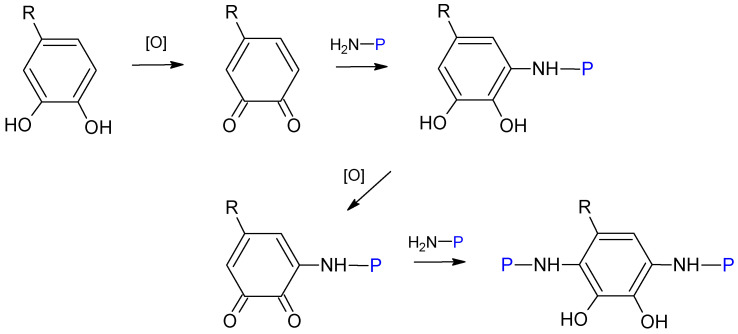
Reactions between a poly-phenolic compound and amine side chains of proteins for cross-linking, where “H2N-P” represents the protein. Modified from the work of [22].

Phenolic compounds with more than one hydroxyl group can induce cross-linking between individual protein molecules [15]. The main chemical pathway is illustrated in Figure 3 and is based on the repeated production of quinone intermediates by oxidation reaction to react with nucleophilic amino acid residues of the protein and form covalent bonds with the phenolic ring [22].

Nataraj et al. [39] studied the cross-linking of horse gram protein by catechol in the presence of sodium periodate to render the films water stability and to enhance the mechanical properties. By the treatment, both the tensile properties and the aqueous stability of the films could be improved, reaching a maximum breaking strength and elongation of 7.3 MPa and 15.3% for 10 and 5% cross-linked films, respectively. The swelling in water decreases with the increase in catechol added. Films with higher concentrations of 20 wt.% catechol displayed significant antibacterial activity against *Bacillus cereus* and *E. coli*, whereas the control horse gram protein film did not show any antibacterial activity. Additionally, the films were stable in water and cell culture media for up to four days suggesting their suitability for tissue engineering and other medical applications.

Santos et al. [54] used non-oxidized and oxidized tannic acid to cross-link zein proteins to enhance the water resistance of films. Water solubility decreased with increasing tannic acid concentration. Additionally, films with oxidized tannic acid-zein showed a higher maximum tensile strength (from 5.6 MPa for the control to 14.0 MPa and 11.2 MPa, respectively) and a lower water vapor permeability than films with non-oxidized tannic acid. The improved mechanical and water barrier properties of films were attributed to the cross-linking effect between oxidized tannic acid and zein. In addition, tannic acid provided the films with a yellowish color and increased opacity.

Complementary, different effects between the non-oxidized and oxidized forms of ferulic acid were also reported by Ou et al. [40] on soy protein isolate (SPI) films. Up to a ferulic acid content of 2 wt.%, the tensile strength of films increased by about 63% (to a final value of 2.6 MPa), while higher concentrations did not further improve the tensile strength. The films cross-linked with oxidized ferulic acid demonstrated further decreased water vapor permeability attributed to the production of free radicals by oxidation and, therefore, higher protein cross-linking capacity. A UV-VIS absorbance shift indicated the formation of a ferulic acid-protein cross-link, also decreasing the oxygen permeability of these films. No values for the mechanical properties of films with oxidized ferulic acid were provided.

Choi et al. [26] investigated the cross-linking effects of small concentrations of different oxidized phenolic substances, including tannic acid, caffeic acid, and green tea extract, on the mechanical and barrier properties of gelatin-based films. Cross-link formation led to higher tensile strength and lower elongation at break, water vapor permeability, and water solubility of films with adequate amounts of cross-linker. Among the various phenolic compounds, the oxidized caffeic acid showed higher mechanical and barrier properties than the other phenolic compounds, increasing the tensile strength from 7.2 to 12.3 MPa for 1 wt.% concentration. However, higher concentrations of phenolic compounds resulted in an increase in elasticity with decreasing tensile strength. The authors described this plasticizing effect by the inhibition of a densely packed structure formation due to remaining phenolic compounds that exist as unbound fractions or phenolic oligomers within the film matrix. Similar observations were made by Zhang et al. [55] for gelatin-based films cross-linked with tannic acid.

#### 3.1.3. Chemical Cross-Linking with Genipin

Genipin, a small molecule with no cytotoxicity issues, has also been investigated as a protein cross-linking agent. The molecule is naturally occurring and is present in fruits of Gardenia jasminoides Ellis [22].

Bigi et al. [56] used genipin to cross-link gelatin films resulting in a drastic decrease in the film elongation and an increased water and heat stability. For 0, 1, and 2 wt.% genipin addition, elongation was measured as 211%, 16%, and 13%, respectively, while tensile strength values kept almost constant around 1 MPa. Cross-linking also reduced the swelling of the films in aqueous solution. Compared to the toxic protein cross-linker glutaraldehyde, extensibility, the enthalpy of denaturation, as well as the swelling properties of the films cross-linked with genipin were quite comparable and showed even higher thermal stability.

Gonzalez et al. [30] evaluated the effect of different genipin concentrations (0 to 10 wt.%) on the properties of glycerol-plasticized soy protein isolate (SPI) films. Cross-linking efficiency and degree could be evidenced by FT-IR analysis. After the cross-linking, the absorption of the band at around 1668 cm^−1^ showed a relative increase due to the formation of new amide linkages between soy proteins and genipin. By the addition of 1 wt.% genipin both the tensile strength and the elongation were increased (to 4 MPa and 46%, respectively). Though, at higher concentrations (7.5 and 10 wt.%), elongation decreased sharply whereas the tensile strength did not significantly change. Even at low genipin concentrations (1 wt.%) the water solubility could be decreased to 35 wt.% (80 wt.% with control). Further, the material acquired a dark blue color and became opaque with increasing cross-linking between protein chains.

#### 3.1.4. Chemical Cross-Linking with Poly-Carboxylic Acids

**Figure 4:**
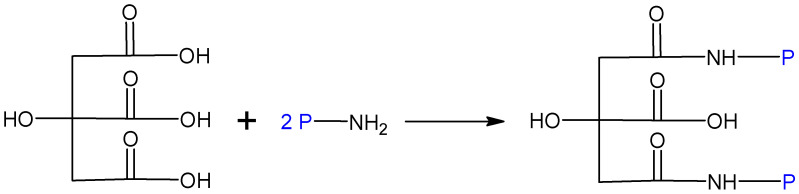
Schematic representation of the cross-linking between citric acid and protein, where “NH2-P” represents the protein.

Poly-carboxylic acids (PCAs) such as citric acid are suitable cross-linking agents for proteins, whereas phosphorous catalysts as sodium hypophosphite are the most effective candidates for catalyzing a reaction with poly-carboxylic acids [57]. A schematic sketch without the catalyst shows Figure 4.

Sakkara et al. [43] extracted proteins from the common legume horse gram, which were used to produce films being cross-linked with citric acid along with the catalyst sodium hypophosphite. Next to an improvement of the tensile strength (from 2.5 to 5.5 MPa), such films cross-linked with citric acid were found to reduce water sorption of films by 50% for 15 wt.% citric acid and exhibited suitable antimicrobial, water vapor, and oxygen barrier properties. The elongation decreased only a little from 25% to at least 20%. FT-IR studies revealed that cross-linking decreases the extent of protein aggregation and interconversion from α-helix to β-sheet appeared.

Yang et al. [58] used citric acid and butanetetra-carboxylic acid as cross-linking agents to increase the strength of zein-based fibers made from dry-spinning. Citric acid at a concentration of 6 wt.% has been reported to cross-link zein protein in the presence of 3.3 wt.% sodium hypophosphite at elevated temperatures for high levels of wrinkle resistance and smooth drying properties durable through repeated laundering in alkaline detergents [36]. After 30 min curing, the produced fibers showed a breaking tenacity of 1 g/d (gram per denier) and a breaking elongation of about 30% compared to 0.38 g/d and 1.7%, respectively, for untreated fibers. These poly-carboxylic acids did not react with proteins at ambient temperatures and thus simplifying the control over the spinning process. Though catalysts that contain phosphorus were related to significant shade changes in dyed fabrics because of their reductive nature [36].

A method of alkali-catalyzed wet cross-linking of plant proteins at low temperatures by carboxylic acids, including malic acid, citric acid, and butanetetra-carboxylic acid, has been reported by Reddy et al. [41]. This approach eliminates the need for phosphorous-containing catalysts or high temperatures for carboxylic acid cross-linking of proteins. Gliadin, soy protein, and zein could all be cross-linked with carboxylic acids that contain two or more carboxylic groups to improve their breaking tenancy by 35.7%, 29.4%, and 54.0%, respectively when compared to un-cross-linked fibers. The cross-linking conditions (temperature, curing time, concentration, pH) can be selected depending on the end-use requirements of the cross-linked materials and the cost and time of cross-linking desired.

### 3.2. Cross-Linking by Radiation

The free radical-mediated grafting method is an alternative method for protein cross-linking to chemical reagents, which are often expensive and sometimes environmentally harmful [15]. One way to approach radicalization in proteins is a treatment with radiation. Molecules capture energy from radiation and convert it into chemical energy so that isomerization is induced by photoexcitation. Proteins that have double bonds and aromatic rings exposed to radiation undergo free radical formation in the respective amino acids, followed by cross-linking reactions [59]. Ultraviolet (UV) and γ-radiation have been shown to induce the formation of protein-protein adducts directly without the use of any external material. Direct coupling of these molecules qualifies radiation as a zero-length cross-linker [49].

Lacroix et al. [60] studied the γ-radiation-mediated cross-linking of milk proteins. Whey protein isolate and calcium caseinate were used pure or in mixed ratios. Puncture strength of films made from whey protein isolate alone increased from 0.08 to 0.14 N/μm when radiation of 32 kGy was applied. With the increasing content of calcium caseinate in the film-forming formulation, the strengthening effect of radiation on the mechanical properties decreased, and films made from only calcium caseinate did not show any improvement. Irrelevant to the mechanical properties, size exclusion chromatography revealed an increase in the concentration of high molecular weight proteins in the film-forming solution. FT-IR and X-ray analysis revealed the formation of inter- and/or intramolecular covalent cross-links.

Schmid et al. [44] observed a significant increase in the tensile strength (from 5.9 to 10.2 MPa) of whey protein-based films by UV irradiation of the protein solution before film formation paired with a yellowing dependent on radiation time. After irradiation, the films showed no significant change in oxygen and water vapor barrier properties or elongation at break. The occurrence of additional molecular interactions could be indicated by a decreased solubility in buffer systems that break disulfide and non-covalent bonds. The authors further argue that packaging materials are often exposed to UV radiation during their storage time, and it may be therefore advantageous that such radiation does not decrease the barrier properties or the elasticity of the product.

For the treatment of soy protein isolate (SPI) with UV radiation, Gennadios et al. [29] complementary reported a linear increase in the tensile strength (from 3.7 up to 6.1 MPa) and a linear decrease in the elongation at break with increasing UV treatment intensity of cast soy protein films plasticized with glycerol. The suitable compatibility of soy proteins for radiation treatment has been explained by a high amount of tyrosine and phenylalanine in their amino acid sequence. Furthermore, they observed increased yellowness of protein-based films after UV irradiation, as observed for whey, soy, and wheat gluten. Besides increased tensile strength, the appearance of immobile bands in electrophoretic patterns suggested further development of covalent cross-links in UV-treated films. Similar observations for the cross-linking of soy protein-based materials were made for γ-irradiation by Lee et al. [35].

The importance of control over radiation-mediated cross-linking of proteins can be demonstrated by the following example. For γ-irradiated gluten films (10 kGy), Micard et al. [38] observed an increase in tensile strength of 32% compared to the control as well as a decrease in elongation, as expected. Though, higher radiation values of up to 40 kGy resulted in the breaking down of covalent linkages and de-polymerization. Lalande et al. [34] made similar observations for gelatin proteins cross-linked by γ-radiation.

### 3.3. Cross-Linking by Heat

Cross-linking of proteins can also be induced by heat. Predominantly, these structural changes are due to the increased polymer chain motility, reshuffling of already existing, as well as the formation of new disulfide bonds [13,18].

For whey proteins, Galani et al. [28] found that temperature increases the exposure of free thiol groups, by β-elimination of cysteine residues, due to α-helix shifts in the protein structure. Polymerization occurred through intermolecular disulfide bond exchange, provided that the temperature is held at above 60 to 65 °C. Additionally, an increase in molecular size was evidenced by gel-electrophorese. The effects of different treatment temperatures (75, 85, and 95 °C) and times (15, 30, and 45 min) on physical, structural, thermal, and morphological characteristics of zein in ethanol-water solution were investigated by Sun et al. [61]. Heat treatment induced significant changes in size and secondary structure of zein, and the specific changing trend was dependent on both the temperature and time.

Hanna et al. [62] studied the modification of glycerol-plasticized soy protein-based films by heat curing. Tensile strength increased from 3.2 MPa for control films with increasing heating time at 80 °C to a maximum of 10.2 MPa after 24 h. Increasing the curing temperature up to 95 °C promoted the observed effect and yielded tensile strength values of 14.3 MPa after 24 h. As expected, the increase in tensile strength came along with a decrease in elongation being linear correlated with heating time. All interactions of films with water were reduced by cross-linking, showing decreases in water solubility, moisture content, and water vapor permeability. In addition, an increase in yellowish color could be evidenced, a characteristic consequence of cross-linking in protein-based films.

Guilbert et al. [38] observed a positive correlation between heating temperature (80, 95, 110, 125, 140 °C) and tensile strength of gluten-based films for short time heat exposure. When the temperature increased from 80 to 140 °C, an increase in tensile strength (2.4 to 7.3 MPa) and a decrease in elongation (391 to 170%) were observed for curing times of 15 min. For shorter treatment times (1.5 min), also at 140 °C still, an increase in tensile strength to 4.2 MPa compared to 1.7 MPa for the control film could be observed. The shorter treatment at high temperature yielded comparable values as those obtained after a 110 °C/15 min treatment.

For the β-elimination of the cystine residues to take place, the presence or absence of a reducing sugar component has been stated to be relevant [18]. The iso-peptide linkages resulting from the reactive intermediates of β-elimination have been identified when no sugar was added before the heat treatment. Otherwise, the heat-induced cross-linking was connected to the reactivity of advanced Maillard reaction products. For example, soy protein isolate and carboxymethyl cellulose were successfully employed to fabricate cast blend films through Maillard-connected cross-linking [63]. FT-IR and 13C NMR spectra confirmed that Maillard reactions occurred forming NCH2 groups, and also, the Maillard reaction was responsible for the dark colors of the composite films. The heat-sealing ability of blend films was superior to that of pure soy protein isolate films. Scanning electron microscopy morphology studies confirmed that in the heat-sealing process, the different polymers melted and diffused through the laminate interface, which strongly depends on the temperature.

Lal and Mhaske et al. [33] developed an efficient protocol for the Maillard reaction-mediated formation of kafirin protein thin film plasticized with PEG-300 and reinforced with different concentrations of oxidized cellulose nanofiber. By the incorporation of only 0.5% of cellulose nanofibers, tensile strength could be increased from 1 to 7 MPa. The cross-linking between the carbonyl group of nanofiber and amine groups of kafirin protein was attributed to the Maillard reaction; this has been explained by the FT-IR spectra. Additionally, an increase in cross-linking between nanofiber and protein resulted in a simultaneous increase in crystallinity and thermal stability.

### 3.4. Cross-Linking by Enzymes

**Figure 5:**
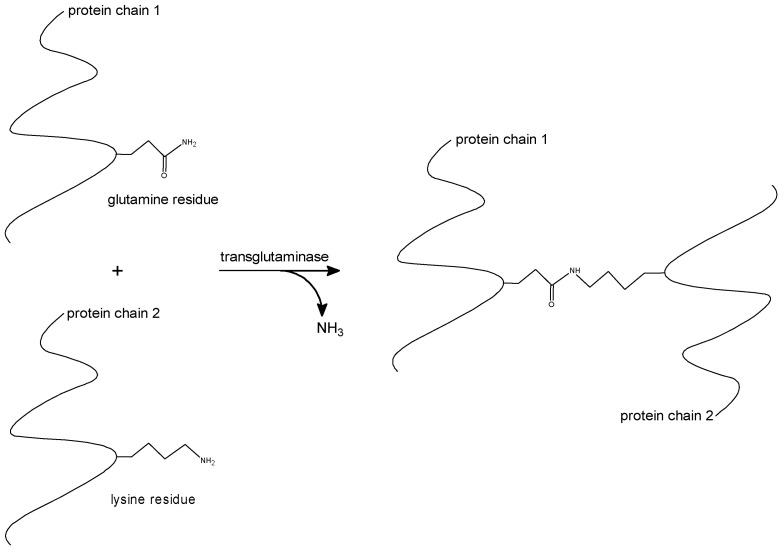
Cross-linking reaction of protein chains catalyzed by the enzyme transglutaminase. Modified from the work of [13].

The use of enzymes to modify the functional properties of proteins has attracted considerable interest since consumers perceive enzymes to be more “natural” than chemicals. Enzymes are also favored as they require milder conditions, show a high specificity, are typically needed only in catalytic quantities, and produce less likely toxic products [36]. Enzymes show great potential as they can mediate the targeted formation of new interactions without denaturation of the protein or insertion of external chemicals. A promising candidate for the implementation in protein films is transglutaminase, widely studied to enhance the cross-linking in proteins by catalyzing cross-linking reactions between glutamine and lysin groups; this results in the formation of intra- and intermolecular cross-linked proteins (Figure 5).

The substrate specificity of the enzyme for the glutamine is high, and only protein-bound glutamines are cross-linked. Most efficient cross-linking occurs in proteins that contain a glutamine residue in a flexible region of the protein or within a reverse turn [36]. In general, proteins with high glutamine content are well suited for enzymatic cross-linking by transglutaminase. For gluten, Schmid et al. [64] reported that the content of ε-(γ-glutamyl) lysine cross-links can be increased by heating, as heating can increase the content of glutamine and lysine residues on the protein surface.

For soy protein isolate films, the relevant functional properties could be improved by treatment with transglutaminase [47]. Mean tensile strength values of cast films with 6 wt.% plasticizer per protein of glycerol, a mixture of glycerol and sorbitol (1:1) and sorbitol, increased by 17%, 20%, and 8%, respectively, up to a maximum of 4.5 MPa after treatment with microbial transglutaminase (4 U/g). In contrast, elongation at break values declined by 34%, 22%, and 73%, respectively. Scanning electron microscopy (SEM) micrographs indicated coarse surfaces and more compact microstructure of cross-sections of films treated with microbial transglutaminase, directly accounting for observed changes in mechanical properties. In addition, treatment with the enzyme increased the surface hydrophobicity by 17% to 56% and simultaneously significantly decreased the moisture content and transparency of films.

The use of transglutaminase (4 U/g) also improved the tensile strength (15 to 17.5 MPa) of zein-based films plasticized with common plasticizer glycerol [37]. The increase in tensile strength has mainly been attributed to the presence of enzyme-induced macromolecular protein complexes in the protein-polymer, as evidenced by the increase in molecular weight. Neither a decrease in elongation, solubility, or water vapor permeability, as characteristic for cross-linking in protein films, was observed.

Blends of proteins treated with transglutaminase have also been investigated. Yildirim and Hettiarachchy [48] used transglutaminase to cross-link whey protein and soy protein for film formation. A positive effect was observed in tensile strength values. The treated films were over two times stronger than control films showing an average of 6.26 MPa. The tensile strength of enzyme-treated films (made from a 1:1 wt.% mixture of soy protein and whey protein) increased to ca. 17.9 MPa. The solubility in water was lower for cross-linked films than for the control.

## 4. Plasticization

A plasticizer is defined as “a substance or material incorporated into a material (usually a plastic or elastomer) to increase its flexibility, workability, or distensibility” [8]. Plasticizers dissolve in the polymer separating chains from each other and thus facilitating molecular movement. The elongation of polymers is affected by the extent of polymer chain associations in the sheet matrix. Plasticizers reduce such associations, decrease tensile strength and increase flexibility. They increase the free volume between chains, introduce more mobility to the polymer, and act as internal lubricants by reducing frictional forces between polymer chains. Plasticizers lower the glass transition temperature [13,65,66].

The complexity of plasticization seems to be a major bottleneck for the improvement of protein-based films. One of the most critical steps to obtain useful protein-based materials is the selection of a plasticizing agent and the determination of its optimal effective concentration. Huber et al. [13] differentiated between internal and external plasticization.

- External plasticizers solvate in between and lubricate the protein chains and also increase the free volume. Because these plasticizers are not chemically bound, they are easily lost by extraction, migration, or evaporation;
- Internal plasticizers chemically modify a protein chain through the addition of substituent groups attached via covalent bonds. They create steric hindrance between the protein chains, leading to increased free volume and improved flexibility.

### 4.1. External Plasticization

Common external plasticizers used in protein films are typically polyols, mono-, di-, and oligosaccharides, as well as fatty acids and phenolic acids [10]. The selection of a plasticizer for a particular polymeric system depends on their compatibility with each other and the desired characteristics of the final product [67]. Based on these criteria, plasticizers were divided into two major categories.

1. Polar plasticizer molecules first bind to protein molecules by hydrogen bonds, interacting with amide groups of the protein. Binding sites do have limited capacities, and clusters can grow around these sites by self-hydrogen bonding when a critical plasticizer concentration is exceeded [20,68]. Polyols as glycerol, polyethylene glycol, or sorbitol are common examples of polar plasticizers;
2. Amphiphilic plasticizers undergo initial hydrophilic adsorption of their polar regions to protein molecules but then also do develop binding to the protein by hydrophobic interactions [20,69]. Phenolic and fatty acids, as well as their derivates, represent common examples.

These two options are discussed in depth below.

#### 4.1.1. Polar Plasticizers

Polyols are hygroscopic molecules generally added to film-forming solutions to prevent film brittleness [70]. From the polyols, glycerol is the most widely applied plasticizer used in the reduction in brittleness, stiffness, and glass transition temperatures of protein-based materials since it is inexpensive, biodegradable, and insensitive to thermal processing [68]. Glycerol forms hydrogen bonds over hydroxyl groups with the amide groups of the protein when they are used as plasticizers [18,66]. Viera et al. [70] explained the plasticizing effect of glycerol by a reduction in internal hydrogen bonds within the protein due to its highly hydrophilic characteristics, and thereby decreasing the internal forces and increasing the intermolecular spacing.

Galietta et al. [71] observed that an increase in glycerol concentration causes an increase in distensibility from 0.3 (at 25 wt.% glycerol) to 0.9 mm (at 40 wt.% glycerol) paired with a decrease in mechanical resistance of whey protein-based films. Increased plasticizer content was also reflected by the observation of a decrease in the glass transition temperature of films, whereas the percentage of solubility in water increased. These mechanical property changes characterize modifications in the three-dimensional organization, decreasing the density and reversibility of intermolecular interactions occurring in the whey protein network and increasing the free volume and chain mobility.

Cuq et al. [12] studied the development of myofibrillar protein-based films from a film-forming solution based on fish mince. Glycerol, sorbitol, or sucrose was incorporated as a plasticizer at various concentrations and induced large decreases in film strength (from 5 to 2 N force at break) and increases in deformation properties (from almost 0 to more than 2 mm) as well as the water vapor permeability. No significant difference was observed in variations of functional properties as a function of plasticizer type when plasticizers were introduced at the same molecular contents. This observation is most likely due to the structural similarities between glycerol, sorbitol, and sucrose.

Bergo and Srobal et al. [72] studied the effects of different glycerol concentrations on the physical properties of pigskin gelatin films. Results from XRD measurements indicated that the plasticizer caused no apparent tendency to re-crystallization in the film structure but to alter other physical properties (e.g., flexibility, interactions between the macromolecule chains, susceptibility to humidity). The mechanical analysis showed a decrease in tensile strength (65 to 10 MPa) and an increase in elongation of films (5% to 40%) for 45 wt.% glycerol with a sharp increase in elongation when concentration increases over 30 wt.%. Additionally, FT-IR spectra showed displacements in the position of the peaks related to glycerol, attributed to hydrogen bonding between the plasticizer and the film structure.

Also, whey protein films were plasticized with different plasticizers (propylene glycol, glycerol, sorbitol, polyethylene glycol (molecular weight of 200 and 400 g/mol), and sucrose) by Krochta et al. [73] to improve their mechanical properties. Glycerol followed by polyethylene glycol with a molecular weight of 200 g/mol is the plasticizer that most efficiently achieved desirable mechanical properties for films. Compared to sorbitol having a similar structure of straight-chain molecules, the smaller size of glycerol and its greater amount of related water increase its effectiveness as a plasticizer. Complementary, the smaller size polyethene glycol was more efficient than the complementary bigger molecule in interacting with protein molecules to decrease elastic modulus and tensile strength values while increasing elongation values. Further, statistical analysis from the data to simulate the plasticizer efficiencies has been carried out. This empirical model fits the changes in the observed mechanical properties for films with up to 30 wt.% plasticizers. The used model values indicated that plasticizer efficiency decreased in the order glycerol, polyethylene glycol with a molecular weight of 200 g/mol, 400 g/mol, sorbitol, and sucrose.

Hedenqvist et al. [74] found that of the tested plasticizers (30 in quantity), glycerol has shown to be the most efficient plasticizer for wheat gluten-based films. The selection of potential plasticizers was based on different molecular weights, polarity, melting, and boiling points, including, among others, many polyols, fatty acids, and amines. Testing of mechanical properties of glycerol-induced films yielded a tensile strength of 2.4 MPa paired with an elongation of 152%. All plasticizer/gluten mixtures were studied at an equal single mass concentration of plasticizer (30 wt.%), and only the short-term mechanical data were analyzed.

The use of di- and tri-ethanolamine as plasticizers permitted Gontard et al. [75] to create wheat gluten-based films with many similar properties to films plasticized with glycerol (i.e., solubility in water, transparency, and water vapor permeability). Concerning the mechanical properties, the use of amines yielded even higher values of elongation, even though at similar glass transition temperatures. These differences were explained by additional binding sites for amines compared to glycerol, where possible ionic bonds in addition to hydrogen bonds contribute to protein-plasticizer interactions. In addition, a modification of the basicity of the casting solution by the presence of amines was mentioned.

Park et al. [76] observed that the dualistic addition of glycerol and polyethene glycol further increases the plasticization effect on zein-based films with a tensile strength of 13.4 MPa and an elongation of 76% for a 50:50 (*v*/*v*) glycerol to polyethene glycol ratio (at a concentration of 0.36 mL plasticizer per gram protein). Additionally, the migration rates of glycerol/polyethene glycol mixtures in zein-based films were slower than that of glycerol alone, slowing down the deterioration of mechanical properties during film storage. Results can be attributed to differences in structure and molecular size between glycerol and polyethene glycol, with the latter containing less hydrophilic functional groups and its bigger molecular weight preventing it from washing out and migration phenomena.

Jiang et al. [77] observed for zein protein that the mechanical properties of films are influenced by the total dose and the ratio of mixed plasticizers simultaneously. When the total dose of plasticizer was 0.35 g/g zein, decreasing the ratio of glycerol to polyethene glycol from 1:0 to 1:1 decreased tensile strength from 5.0 MPa to less than 3.8 MPa and increased elasticity at break from less than 5% to more than 50%. FT-IR measurements showed that these films had lower tightness of inter-a-helix packing and higher content of β-sheet. Therefore, the improvement of mechanical properties could be attributed to the structural change in zein molecules. Higher plasticization (elongation at break of more than 200%) was achieved when a mass ratio of 1:1 of glycerol to polyethylene glycol was increased to a concentration of 0.45 g plasticizer per g zein. Nonetheless, such excessively high doses of the additives made films stick to each other probably because of the migration of the plasticizer.

The nature of the interaction between protein molecules and glycerol is weak (H-bonding), and excess glycerol easily migrates through the film matrix [18,65,78]. Polyols as glycerol interact non-covalently with the polymer matrix. These plasticizers undergo migration toward the material surface that, in the end, lead to physical instabilities (material aging) and increased stiffness and brittleness [10]. To encounter the leaching of plasticizers by the creation of a barrier, several techniques, such as coating of polymer surfaces and plasma surface-treatment, have been explored. Anyhow, further processing makes the product more expensive, more complicated to produce, and sometimes even decreases the plasticizer effectiveness [79].

Next to the migration of polar plasticizers, the increased hygroscopy of respective films is mentioned as a concern for their application in plastics. Han et al. [80] wanted to produce transparent zein films with glycerol as the plasticizer. The high degree of polarity results in hygroscopic properties of glycerol, which are then transferred to the material. Glycerol could easily absorb water from the air to form small droplets on the film surface and in internal voids. The light scattering of these water droplets not only reduced film transparency but the remaining water in the film also affected characteristics of the film because water can act as a plasticizer for proteins. Robin et al. [81] reported that in the case of gluten, zein or casein glycerol stands out as the most used plasticizer but also pointed out that the final material absorbs water, which leads to a plasticized material with decreased mechanical properties. Chen et al. [68] reported that water interacts with protein chains and influences the protein structure. Thus, film moisture content, as affected by the relative humidity of the surrounding environment, would largely affect functional properties. The application of polar plasticizers does increase the natural hygroscopic nature of proteins, and such produced materials will always be susceptible to moisture-dependent changes in mechanical as well as barrier properties.

#### 4.1.2. Amphiphilic Plasticizers

The water barrier capability of protein films can be improved by the use of less polar, amphiphilic plasticizers. In this context, fatty acids have received substantial attention. Vegetable oils derived from soybean, linseed, palm, and castor bean contain fatty acids of various kinds and can potentially be used as plasticizers after chemical modifications [81]. Furthermore, phenolic acids were reported to interact with proline-rich proteins via hydrophobic interactions and hydrogen bonding, making them appropriate candidates for protein plasticization [82]. In general, proteins containing a high amount of hydrophobic amino acids have been noted to provide an excellent opportunity to use pure phenolic or fatty acids because respective materials are prepared in aqueous ethanol, an effective solvent for most of these compounds [83].

A series of saturated fatty acids with different carbon chain lengths (from 6 to 14 carbons) was added to wheat gluten film by Guilbert et al. [84] to evaluate its plasticizing effect and to improve the water barrier. Plasticization was successful, evidenced by a decrease in the glass transition temperature from 180 °C to a minimum of less than 60 °C, and the relation between aliphatic chain length of fatty acids (6 to 10) and compatibility with the gluten protein could be defined. Increasing the molecular weight of saturated fatty acids reduces the plasticization effect. Smaller molecules were more easily incorporated into the protein matrix and exhibited a more efficient plasticizing effect. The compatibility limit in concentration decreased with increasing carbon chain length following an exponential law. Beyond this limit, a fatty acid exudation was observed in the material. The plasticizer is then not any more compatible with wheat gluten, and the corresponding material becomes heterogeneous. The same anti-proportionality between fatty acid length and plasticization efficiency was observed by Krochta and Sothornvit [85] for fish myofibrillar protein films.

Lai et al. [86] reported the tensile properties, water absorption, and microstructure of zein sheets plasticized with palmitic and stearic acids. The tensile strength of zein sheets was found to increase with the addition of low levels of fatty acids from 5 to almost 15 MPa for both plasticizers. However, beyond a critical point, the strength decreased with the further addition of fatty acids because of their limited compatibility with the protein. Water absorption decreased continuously with increasing fatty acid content. This is in accordance with Martins, reporting that the resistance of a film to water vapor is inversely proportional to the polarity of the inserted lipids [5]. Though, Lai et al. [86] found that palmitic acid (C 16) exhibits a better plasticization effect over stearic acid (C 18) due to its lower melting point and, therefore, better dispersion throughout the zein film. Even stearic acid is less polar. It has a higher melting point than palmitic acid; thus, it may have re-solidified faster than palmitic acid during processing. Solidification of stearic acid was found to prevent its complete mixing with zein resulting in less effective plasticization.

Concerning the solidification of higher molecular weight fatty acids, similar observations were made by Fulcher et al. [87], studying the incorporation of fatty acids from C 14 to C 22 (myristic, palmitic, stearic, arachidic, and behenic acids) as plasticizers in whey protein isolate (WPI) films. All formulations contained similar percentiles of small crystals, with very large globular crystals measured among arachidic and behenic acids. Such increased heterogeneity of the film matrix lowers the mechanical properties and should be avoided.

Padua and Santosa [65] investigated the effect of oleic acid and linoleic acid on mechanical properties and water absorption of zein sheets. Plasticization increased elongation and decreased water absorption of zein sheets. Tensile strength of oleic acid-plasticized sheets decreased from 9.4 to 2.2 Mpa as plasticizer level increased from 0.5 to 0.9 g of oleic acid per g of zein. A maximum elongation for all films of 45% was observed at 0.6 to 0.7 g of oleic acid per g of zein. Linoleic acid was more effective than oleic acid at reducing water absorption of zein sheets, possibly due to linoleic acid polymerization, which may have filled pores and gaps in the structure, preventing it from swelling. Nevertheless, a limited capacity of the zein structure to absorb fatty acids (for oleic acid beyond 0.9 or linoleic above 0.7 g per g of zein) have been reported. Excess fatty acid weakened the structure, lowering elongation, toughness, and energy to the breakpoint.

Also, the addition of fatty acids (i.e., lauric, myristic, palmitic, oleic) to soy protein isolate film-forming solutions was reported to cause a substantial decrease in water vapor permeability as well as a plasticizing effect translated into a much higher elongation at break [88]. Oleic acid, as only unsaturated fatty acid, developed the highest plasticization strength with a maximum of 228% elongation for 10 wt.% oleic acid compared to 70% elongation in the control. This has been attributed to the mobility of oleic acid within the film structure imparted by the double bond. As another concern regarding protein-lipid films is the potential susceptibility of plasticized films to lipid oxidation.

Wei et al. [20] reported that during a 6 week storage period of oleic acid-plasticized zein films in a light stability cabinet at room temperature, discoloration and increased brittleness in the films were observed. This was traced to the migration and oxidation of plasticizer oleic acid used in this film and was measurable by weight loss. In addition, the development of off-odors has been reported when zein films were plasticized with 40 to 50 wt.% oleic acid [89]. However, Han et al. [90] mentioned that antioxidants such as butylated hydroxytoluene (BHT) or butylated hydroxyanisole (BHA) can be used to prevent lipid oxidation of protein-based films plasticized with fatty acids.

Hager et al. [91] observed that different phenolic acids may present different effects on gluten-based films. While tannic acid, as explicated in Section 3.1.2, acted noticeably as a cross-linker in gluten films, gallic acid acted instead as a plasticizer, increasing elongation of films from 205% to a maximum of 297% for the addition of 10 wt.% gallic acid. The differences were explained by differences in polarity between the two phenolic acids. Gallic acid is overall more polar than tannic acid and promotes the replacement of protein intra-hydrogen bonds with hydrogen bonds between the several OH-groups of gallic acid and hydrophilic sides of the protein. Additionally, phenolic compounds with larger molecular sizes (as tannic acid) have more potential binding sites, which also relates to their higher capacity of binding peptide chains at more than one point.

Yemenicioğlu and Arcan et al. [92] incorporated several phenolic acids (gallic acid, p-hydroxybenzoic acid, ferulic acid) into zein-based films to achieve flexible bioactive packaging materials. Compared to plasticizing with glycerol, the incorporation of phenolic acids could not only reduce the brittleness and increase the flexibility (showing elongations between 135% and 189%) but also provide protein films with antioxidant and antimicrobial characteristics.

### 4.2. Internal Plasticization

Not much research has been published on the internal plasticization of protein-based film to target the increase in mechanical properties. Only some modification methods are reported to achieve covalent bonding between a protein and the respective plasticizer.

Wheelwright et al. [93] studied the internal plasticization of zein by esterification with methanol via a covalent bonding between the plasticizer and protein. Their work aimed to develop a method for long-time plasticization of protein-based films without migration of external plasticizer molecules. Authors successfully modified zein by the formation of the methyl ester, principally via the amide groups of the protein, evidenced by FT-IR spectroscopy and differential scanning calorimetry. By this modification, the glass transition temperature was reduced from 162 to 143 °C. Authors further mention that by optimized reaction conditions, a greater extent of methylation might be possible but point out that hereby a first step of functionalizing a method for internal plasticization of zein was achieved.

Another common chemical derivatization scheme next to esterification is acylation (e.g., acetylation and succinylation). A protein considered for an acylation reaction requires the existence and exposure of nucleophilic amino acid residues (e.g., an amino or phenol group). Respective acylating agents (e.g., activated acid anhydrides) contain a carbonyl group to react with. [59].

Shi et al. [94] investigated the modification of zein with lauryl chloride through an acylation reaction with lauryl chloride. Such treatment yielded a by about seven-fold elongation at a break value of 302% compared to 47% for the control. Modified zein-based films also showed a decrease in the glass transition temperature, while the tensile strength stayed almost constant, around 3 Mpa. Atomic force microscopy images showed that surfaces of modified zein films became more uniform but also more hydrophobic. Moreover, the infrared spectra of the zein samples revealed a difference in molecular structures between modified and pure zein, and results from gel-electrophorese analysis indicated an increased molecular weight of proteins.

Bräuer et al. [95] integrated 4 mmol/g palmitoyl groups into wheat gluten as well as into soy protein for preparation of biodegradable protein materials by extrusion. For improved processability and mechanical performance, the addition of 10% glycerol has been proposed. In most cases, the extruded products showed a high brittleness and low mechanical stability independent of the substitution pattern. Thus, the processability of acylated plant proteins by extrusion was demonstrated, but the mechanical properties of extruded products have still to be improved.

## 5. Conclusions

With the aim to increase the mechanical properties of protein-based materials, several researchers have applied and analyzed plasticization and cross-linking methods. The aim of cross-linking is to increase the tensile strength by the formation of intermolecular linkages. From the chemicals used for cross-linking, the use of aldehydes has been assessed for decades. As they are considered to be toxic, the related research activities have been consequently reduced. Biologically derived aldehydes enable a safe use for biologically benign materials, but yet with moderate efficiency. Only for dialdehyde alginate, not for dialdehyde starch, the tensile strength could be increased. The polyhydric and polymeric nature of these agents might lead to plasticization due to their hygroscopy and sterically hindrance, respectively, introduced into the polymer matrix. Poly-phenols such as ferulic acid, catechol, or tannic acid were also applied as cross-linker. For suitable effectiveness, oxidation of diphenol moieties is required, increasing the cross-linking capacity by radicalization. For concentrations higher than 1 to 2 wt.% plasticization of treated materials occurs, based on limited binding sites on the protein and self-polymerization of the cross-linker inhibiting the formation of a densely packed structure. The use of genipin as a cross-linker showed only moderate efficiency with compatibility issues and the introduction of dark color into the material. Cross-linking with poly-carboxylic acids (e.g., citric acid, butane-tetracarboxylic acid) shows high potential to increase the strength of protein-based materials. These cross-linker do not interact with proteins at ambient conditions, which simplifies the control over the cross-linking process. However, such a procedure requires the addition of phosphorus catalysts being highly reductive and potentially toxic to the environment. An alternative alkali-catalyzed method has been presented, which eliminates the need for a phosphorous catalyst but requires further optimization. In general, low to moderate concentrations of cross-linking agents successfully increased the tensile strength, but higher effectiveness requires some kind of mediation to increase the compatibility between protein and cross-linker.

Irradiation (γ- and UV radiation) of protein-based materials has been successfully used as a zero-length cross-linker, increasing the tensile strength without the addition of any cross-linking agents. While for soy protein, both γ- and UV radiation increased the tensile strength, for casein, only γ-irradiation did. Individual proteins exhibit different degrees of response to radiation due to varying amino acid compositions and molecular structures. Tyrosine and phenylalanine as aromatic amino acids in soy protein are more suspicious to radiation, as the treatment induces the radicalization of aromatic rings required for the cross-linking. In addition, changes in the processes from conformational change and cross-linking to covalent bond cleavage in the backbone and side chains of proteins appear with higher radiation doses. Irradiation influences all molecules within the system, resulting in different material properties for the same protein and irradiation source but with an additive such as a plasticizer. Cross-linking was most effective by heat treatment. The nature of cross-linking mechanism induced by heat depends on the presence or absence of reducing sugar in the system (e.g., glucose, cellulose). In the absence of sugar, the reaction results from the beta-elimination of cysteine residues, whereas otherwise, the Maillard reaction between protein and sugar is dominant. Though, each reaction’s dynamic is fast and very sensitive to temperature and time of exposure to it. Thus, it requires controlled conditions specific to each protein species, while the thermal stability of proteins is also dependent on other molecules in the system. Proteins cross-linked with enzymes yield in “natural” and non-toxic materials with a moderate increase in tensile strength but require further investigations for the application in specific solvent systems and possible immobilization techniques to ensure higher effectiveness at moderate costs. Next to higher tensile strength and barrier properties, most cross-linked materials present increased thermal stability, decreased degradation rates, transparency, less swelling, and water solubility as well as antimicrobial activity.

To increase the flexibility of protein-based materials, several researchers have reported the application of natural and/or biodegradable plasticizers, which reduce polymer chain-to-chain interactions by binding to the protein, distributing throughout the polymer matrix, and therefore increasing the free internal space. External plasticizers, binding over weak interactions, are differentiated based on their polarity. Polyols, accounting for polar und fatty acids and phenolic acids for less polar, amphiphilic ones. Glycerol has been condensed as the model plasticizer, showing the highest plasticization efficiency but also concerned about its hygroscopicity transferred to the material. Mixtures of glycerol with less polar plasticizers with higher molecular weight as several polyethylene glycols yielded synergistic properties. In general, because of the hygroscopicity of biopolymers and plasticizers, the moisture content of films and coatings is affected by the ambient conditions. Generally, water is considered the most powerful plasticizer for hydrocolloid-based films and coatings. The use of hydrophilic plasticizers may even increase the water content of the matrix as it can facilitate water absorption from air, which confers flexibility to the matrices with decreased stability at high relative humidity. On the other hand, the less polar amphiphilic plasticizers as fatty and phenolic acids decrease the water sorption properties of materials but also show a lower plasticization effect and limited compatibility with the protein. Concerning their structure, higher compatibility and plasticization efficiency is achieved for shorter fatty acids, being more polar. The compatibility also depends on the specific protein and its hydrophobicity, with some proteins being suitable for plasticization with longer fatty acids, which results in more overall hydrophobic materials. Additionally to the polarity, the melting temperature influenced by the molecular weight and saturation level of fatty acid was shown to play an important role as molecules with higher melting temperatures tend to recrystallize inside the material and weaken the molecular structure of the matrix. Proteins have limited binding sides, and excess fatty acids result in weakened structures. A major concern about the use of fatty acids is their potential oxidation, which weakens the performance of the plasticized materials over time and can develop off-odors. Research on phenolic acids for plasticization of protein-based materials is rare but has been approved for gallic acid, for example, which may apply additional antioxidant and antimicrobial characteristics to the plasticized material.

In general, external plasticization, induced by weak hydrogen bonding and hydrophobic interactions, does not require harsh reaction conditions and can be mostly achieved by simply mixing the components at room conditions without the addition of auxiliary chemicals. A drawback of these weak interactions is their instability over time, which results in the long-term migration of plasticizer molecules throughout the matrix onto the surface, decreasing the material flexibility and can cause stickiness of such. Interferingly, migration and plasticization electiveness increase both for plasticizers with small size and high polarity. So that the use of external plasticizers should be chosen according to the desired functionalities of the material, where both a high plasticization efficiency and low water interactions cannot be achieved with a single plasticizer. The synergistic plasticizing effects of mixed plasticizers could improve plasticizing efficiency and save additives and costs. A way to accomplish stable plasticization without migration is internal plasticization, where plasticizer molecules are covalently bound to the protein, therefore being part of a new polymer. For example, acylation with fatty acid chlorides acts by a covalent binding mechanism and was proven for a high plasticization of protein-based materials without losses of tensile strength. The development of new materials based on the reactive properties of the functional groups of protein’s amino acids is a very specific and purposeful route to protein stable plasticization but requires more research for the development of specific binding mechanisms and resulting protein structural reconfigurations.

## 6. Discussion

Other than synthesized polymers (bio or fossil based), proteins are hetero-polymers consisting of 20 different monomers (amino acids). This complexity provides protein-based materials with a high molecular binding capacity but requires targeted modification of specific bonds to develop defined molecular structures, which contribute to the required material properties. Because basic information is essential to design films with specific molecular structures and mechanical properties, a systematic approach for understanding the binding mechanism is essential.

Cross-linking requires covalent bonding between the protein and the cross-linker at two sides. Therefore, the specific binding sites need to be existent as well as exposed to both parties. Reactivity of a chemical is conducted with its toxicity as for aldehydes so that if non-toxic chemicals are used, they require some kind of mediation to be reactive toward the protein and interact by covalent bonding. For phenolic compounds, oxidation yields in electrophilic quinones, which then react with nucleophiles (mainly amino or sulfhydryl side chains of proteins). The use of poly-carboxylic acids requires the addition of a catalyst (poly-carboxylic acids) to reach suitable efficiencies. On the other side, the protein must contain compatible amino acid residues with either amino or carboxyl group groups, and additionally, these need to be exposed and not buried inside of protein structural elements. In addition, for physical cross-linking methods, the protein’s specific amino acid sequence and structure is important. For radiation-induced cross-linking, the presence and exposure of aromatic residues (e.g., tyrosine, phenylalanine) determines the extent of potential cross-linking for heat curing the amino acid cysteine is required for cross-linking reactions.

Plasticization in its common form by external plasticization functions over hydrogen bonding between the protein and the plasticizer, requiring hydroxy groups. The higher the number of hydroxy groups in the plasticizer, the higher is its effectiveness for plasticization due to increased hydrogen bonding and cluster-forming capacity. Though, the high polarity of such molecules increases the overall hygroscopy of protein-based materials, which has been addressed as a major drawback for most applications of such products. Additionally, the energy of hydrogen bonding is low, and such weak interactions can be disrupted by time or interactions with water, leading to migration of the plasticizer out of the polymer matrix and yielding decreased plasticization. Amphiphilic plasticizers such as fatty or phenolic acids can reduce the overall hygroscopy of the material, though with lower plasticization effectiveness. However, their interaction with proteins also relies on weak forces (hydrogen bonding and hydrophobic interactions). Additionally to the potential migration and hygroscopy of external plasticizers, they come with another major drawback as their effectiveness always includes a decrease in tensile strength of the material. When a binding site on the protein is already occupied, clusters can grow around these sites by hydrogen bonds or hydrophobic interactions, respectively, between multiple plasticizer molecules. The method of internal plasticization does circumvent the issues of migration, hygroscopy, and loss of tensile strength by targeted covalent bonding under controlled reaction conditions. Similar to the methods of cross-linking, this approach requires the same information about protein primary and secondary structure to optimize the effectiveness of modification.

Proteins vary in their three-dimensional structure as well as their surface compositions. A particular amino acid, and binding partner for plasticizer molecules, may occur both buried in a protein’s interior and exposed on the protein’s surface. This duality may or may not be true for another protein and environment. It has also been observed that plasticization and cross-linking induce a shift of these protein structural elements. An increase in β-sheet structure with an increase in plasticizer content is ascribed as a response to the interference caused by the plasticizer (less order), while the increase in α-helical content with cross-linking is due to the increase in order from cross-linking. The secondary structure whereas is of special interest to researchers because it has been found that functional properties, such as mechanical and gas permeability properties, are related to protein secondary structure. Due to the high amount of self-hydrogen bonding, α-helical structures are the most compact secondary structures. These interactions result in less exposed hydrophilic sites and altogether higher hydrophobicity. Additionally to the high amount of intramolecular hydrogen bonds, the helical structure also allows for a denser packing of the peptide backbone compared to the elongated and flat structure of β-sheets. During the manufacturing of protein-based materials, it is of great importance to understand how the structure is changing during these processes and whether new bonds are forming or the existing bonds are being disrupted. The amount of each secondary structure varies for the same protein depending on such factors as include physical (heating, shearing, hydrostatic pressure or irradiation), chemical (alkylation, acylation, acetylation or pH alteration), and biochemical ones (enzymes). Such extrinsic factors induce the protein denaturation, promoting protein unfolding and the exposure of functional groups followed by newly induced chain associations to stabilize the three-dimensional network. To design explicit networks and induce specific material properties, control over the structural dynamics from the raw material, over-processing up to the final product is essential.

Several methods for the improvement of functional properties have been presented, with the targeted induction of covalent bonds being most promising, but in general, the limited knowledge and control over such binding reactions and their influence on protein structure currently limit the potential of protein-based materials. Therefore, the use of appropriate analytical methods is required to identify and track protein interactions and structure during modification.

## Appendix A

**Table A1:** Summary of mechanical properties of protein-based films after cross-linking modification. Extended data, including error intervals and a more detailed description of methods, are provided in the respective publications.

Protein Source	Cross-Linking Treatment	Tensile Strength	Elongation	Testing Other Than Mechanical	Reference
Pig skin gelatin	Genipin	1.0 MPa-	211%-	DSC, determination of cross-linking degree, release properties, swelling	[56]
	0.07 wt.%	1.2	60		
	0.15 wt.%	1.0	34		
	0.33 wt.%	1.0	19		
	0.67 wt.%	1.2	16		
	1.00 wt.%	1.2	16		
	1.50 wt.%	1.1	17		
	2.00 wt.%	1.0	13		
Soy protein isolate (with 50 wt.% glycerol)	Genipin	3.2 MPa-	22.5%-	Determination of cross-linking degree, opacity, moisture content, swelling ratio, WVP, SEM, IR	[30]
	0.1%	3.3	26.7		
	1.0%	4.2	45.8		
	2.5%	4.5	36.9		
	5.0%	4.6	12.1		
	7.5%	4.5	3.2		
	10.0%	4.6	2.8		
Horse gram protein	Citric acid	2.2 MPa-	25%-	WVTR, OTR, FT-IR, SEM, antioxidant and antimicrobial activity	[43]
	5%	4	23		
	10%	4.6	21		
	15%	5.5	20		
Gelatin	Alginate dialdehyde	1.2 MPa-	306%-	X-ray, swelling, determination of cross-linking degree	[51]
	1 wt.%	2.2	275		
	3 wt.%	2.7	232		
Gelatin (with 30% glycerol)	Dialdehyde starch	3.9 MPa-	96.6%-	Thermogravimetric analysis, opacity, water uptake, WVP, OP, SEM, soil degradation	[52]
	5%	2.7	158.2		
	10%	3.5	111.8		
	30%	1.1	55.3		
Horse gram protein	Catechol	2.9 MPa-	7.1%-	FT-IR, HPLC, water sorption, thermo gravimetric analysis, antioxidant studies, antimicrobial studies	[39]
	5%	4.6	4.6		
	10%	7.3	4.5		
	15%	0.9	1.9		
	20%	1.1	1.3		
Corn zein	Tannic acid	5.58 MPa-	2.16%-	FT-IR, opacity and color, SEM, WVP, water solubility	[54]
	4%	7.43	0.71		
	8%	11.17	0.80		
	Oxidized tannic acid				
	4%	9.32	0.84		
	8%	13.97	0.81		
Soy protein isolate with 60 wt. % glycerol	Ferulic acid	1.60 MPa	156.3%-	WVP, oxidant studies, UV-VIS spectrometry	[40]
	1 wt.%	2.07	167.0		
	2 wt.%	2.60	165.3		
	3 wt.%	2.44	166.5		
	4 wt.%	2.17	155.4		
Gelatin with turmeric acid	Oxidized tannic acid	7.3 MPa-	26.7%-	FT-IR, WVP	[26]
	1 wt.%	8.3	24.9		
	2 wt.%	10.3	25.8		
	3 wt.%	9.7	27.4		
	Oxidized caffeic acid				
	1 wt.%	12.3	25.7		
	2 wt.%	11.8	25.6		
	3 wt.%	10.2	25.8		
	Oxidized reen tea extract				
	1 wt.%	7.3	25.1		
	2 wt.%	7.9	25.0		
	3 wt.%	9.8	22.9		
Milk proteins	γ-irradiation (32 kGy)	(N/um)	-	WVP, FT-iR, X-ray, transmission electron microscopy (TEM), biodegradation, SEC	[32]
	Whey protein isolate	0.08–0.14			
	Calcium caseinate	0.04–0.04			
Whey protein isolate	UV radiation (J/cm^2^)	5.9 MPa-	58%-	Color, light transmittance, OTR, WVTR, surface tension	[44]
	2.3	7.2	64		
	10.2	8.2	47		
	19.0	8.5	54		
	31.4	10.2	40		
Soy protein isolate	γ-irradiation (kGy)	1.6 MPa-	248.7%-	WVP, SEM, color studies, SDS-PAGE	[35]
	4	1.7	204.4		
	16	2.1	196.7		
	32	2.8	190.9		
	50	3.2	164.2		
Soy protein isolate	UV radiation (J/m^2^)	3.7 MPa-	124%-	SDS-PAGE, WVP, color studies	[29]
	5	4.5	127		
	25	4.8	118		
	40	5.2	106		
	52	5.3	113		
	78	5.5	92		
	105	6.1	85		
Wheat gluten	γ-irradiation (kGy)	2.1 Mpa-	384%-	WVP, water solubility, color studies, heat treatment, aging	[38]
	10	3.0	261		
	20	2.6	344		
	40	2.7	297		
Soy protein isolate	Heating time at 80 °C	3.2 MPa-	112%-	Moisture content, water solubility, WVP, color studies	[62]
	2 h	5.1	75		
	6 h	7.1	70		
	14 h	8.7	40		
	24 h	10.2	30		
	Heating time at 95 °C				
	2 h	7.8	63		
	6 h	9.7	58		
	14 h	13.1	22		
	24 h	14.3	25		
Wheat gluten	Heat treatment (°C)	1.7 MPa-	501%-	WVP, water solubility, color studies, radiation treatment, aging	[38]
	80 (15 min)	2.4	391		
	95 (15 min)	2.5	386		
	110 (15 min)	3.1	327		
	125 (15 min)	6.3	275		
	140 (15 min)	7.3	170		
	140 (1.5 min)	4.2	326		
Soy protein isolate with 60 wt.% plasticizer	Control	(MPa)	(%)	WVP, moisture content, lipid barrier property, transparency, surface hydrophobicity, SEM	[47]
	Glycerol	2.2	160		
	Glycerol/sorbitol (1:1)	2.6	102		
	Sorbitol	4.2	102		
	MTGase-treated (4 U/g)				
	Glycerol	2.6	106		
	Glycerol/sorbitol (1:1)	3.1	80		
	Sorbitol	4.5	27		
Corn zein	Control	(MPa)	(%)	Molecular weight distribution, WVP, moisture content, solubility, SEM	[37]
	Ethylene glycol	22	3.5		
	Propylene glycol	17	3.7		
	Glycerol	15	3.7		
	Sorbitol	20	3.4		
	MTGase-treated (4 U/g)				
	Ethylene glycol	27	3.7		
	Propylene glycol	20	4.1		
	Glycerol	18	3.5		
	Sorbitol	24	3.7		
Gelatin + casein (with 25 wt.% glycerol)	Control (gelatin:casein)	(MPa)	(%)	WVP, SEM, SDS-PAGE	[25]
	100:0	12	9		
	75:25	17	27		
	50:50	23	27		
	25:75	24	22		
	0:100	36	11		
	MTGase-treated (10 U/g)				
	100:0	12	18		
	75:25	17	58		
	50:50	21	32		
	25:75	23	32		
	0:100	37	18		
Whey + soya protein (with 1.25% glycerol)	Control (whey:soya protein)		-	Solubility, enzymatic hydrolysis, WVP, SDS-PAGE	[48]
	100:0	5.64			
	50:50	7.61			
	0:100	6.26			
	MTGase-treated (20 U/g)				
	100:0	12.53			
	50:50	16.41			
	0:100	17.86			

**Table A2:** Summary of mechanical properties of protein-based films after plasticization modification. Extended data, including error intervals and a more detailed description of methods, are provided in the respective publications.

Protein Source	Plasticizing Treatment	Tensile Strength	Elongation	Testing Other Than Mechanical	Reference
Whey protein (+formaldehyde and CaCo_3_)	Glycerol	(N/mm)	(mm)	Water solubility, rheometer, dynamic mechanical thermal analysis	[71]
	25 wt.%	18	0.3		
	40 wt.%	7	0.9		
Fish myofibrillar		5 N-	0.3 mm-	WVP	[12]
	Glycerol (40 wt.%)	2.8	2.5		
	Sorbitol (80 wt.%)	2.9	0.7		
	Sucrose (150 wt.%)	2.9	0.4		
Pigskin gelatin	Glycerol	65 MPa-	<5%-	FT-IR, XRD, microwave insertion loss	[72]
	30 wt.%	30	5		
	45 wt.%	10	40		
Whey protein	(0.34–0.64 M)	37.5 MPa-	0%-	Statistical analysis software (SAS)	[73]
	Glycerol	5–16	11.4–76.5		
	Sorbitol	2.7–10.1	24.8–65.9		
	Sucrose	1.7–9.7	30.3–89.4		
	PEG 200	1.8–6.5	41.7–77.1		
	PEG 400	0.7–2.9	25.5–32.3		
Wheat gluten	(All 30 wt.%)	(MPa)	(%)	Rheological measurements	[74]
	Glycerol	2.4	152		
	Ethylen glycol	21.5	2		
	Diethylene glycol	3.4	66		
	Triethylene glycol	2.8	99		
	Tetraethylene glycol	4.4	34		
	1.2-propanediol	13.2	2		
	1.3-propanediol	9	3		
	1.4-butanediol	9.9	2		
	Diethanolamine	4.5	16		
	Triethanolamine	5.7	78		
	Trimethylpropane	2.9	104		
Wheat gluten	Glycerol	(MPa)	(%)	WVP, dynamic mechanical thermal analysis (DMTA), opacity, weight loss in water	[75]
	10% (58% RH)	12.1	4		
	10% (98% RH)	0.4	11		
	20% (58% RH)	2.6	22		
	20% (98% RH)	0.9	30		
	Diethanolamine				
	10% (58% RH)	7.5	9		
	10% (58% RH)	1.0	61		
	20% (58% RH)	4.1	125		
	20% (98% RH)	0.8	58		
	Triethanolamine				
	10% (58% RH)	10.1	6		
	10% (98% RH)	1.3	103		
	20% (58% RH)	5.3	114		
	20% (98% RH)	0.8	54		
Corn zein (also for wheat gluten)	Glycerol:PEG	(MPa)	(%)	Storage test, WVP	[76]
	at 0.36 mL P/g protein				
	100:0	8.4	3		
	75:25	9.6	7		
	50:50	13.4	76		
	25:75	8.4	89		
	0:100	9.5	94		
	at 0.28 mL P/g protein				
	100:0	18.1	3		
	75:25	20.2	6		
	50:50	22.8	18		
	25:75	21.8	19		
	0:100	22.1	23		
	at 0.20 mL P/g protein				
	100:0	26.2	2		
	75:25	32.7	3		
	50:50	35.5	7		
	25:75	30.9	9		
	0:100	33.2	10		
Corn zein (also for gly and PPG and gly and PTMG) and also in concentrations of 25, 33, 66, and 75%	Glycerol:PEG	6 MPa-	1.4%-	FT-IR, SEM, XRD, DSC, WVP	[77]
	at 0.35 g P/g protein				
	100:0	5	<10		
	50:50	2.2	120		
	0:100	4	30		
	at 0.4 g P/g protein				
	100:0	4.6	<10		
	50:50	1.5	330		
	0:100	0.5	150		
	at 0.45 g P/g protein				
	100:0	3.5	<10		
	50:50	1.5	350		
	0:100	0.5	300		
Corn zein	Palmitic acid	5.2 MPa-	0.5%-	Water absorption, SEM	[86]
	25 wt.%	10.6	0.6		
	50 wt.%	14.6	1.0		
	75 wt.%	11.6	0.8		
	100 wt.%	9.9	0.8		
	Stearic acid				
	25 wt.%	13.6	0.9		
	50 wt.%	6.9	0.5		
	75 wt.%	10.5	0.8		
	100 wt.%	8.0	0.6		
Corn zein	Oleic acid	(MPa)	(%)	Water absorption	[65]
	50 wt.%	9.4	5.9		
	60 wt.%	5.9	43.6		
	70 wt.%	4.2	46.9		
	80 wt.%	3.4	17.7		
	90 wt.%	2.0	11.0		
	100 wt.%	2.2	7.5		
	Linoleic acid				
	60 wt.%	5.9	14.5		
	70 wt.%	4.7	27.6		
	90 wt.%	4.6	12.3		
Soy protein	Lauric acid	4.4 MPa-	70.1%-	WVP, color	[88]
	10 wt.%	1.9	125.8		
	20 wt.%	1.0	26.2		
	30 wt.%	-	-		
	Myristic acid				
	10 wt.%	0.4	41.0		
	20 wt.%	0.5	42.0		
	30 wt.%	0.6	19.8		
	Palmitic acid				
	10 wt.%	1.2	39.0		
	20 wt.%	0.8	18.3		
	30 wt.%	-	-		
	Oleic acid				
	10 wt.%	2.2	228.1		
	20 wt.%	1.7	149.2		
	30 wt.%	1.6	140.8		
Wheat gluten	Gallic acid	1.1 MPa-	204.9%-	WVP, SEM	[91]
	1%	1.0	196.4		
	2%	0.9	187.7		
	5%	0.8	238.2		
	10%	0.5	296.8		
Gelatin	Tannic acid	84 MPa-	9.8%-	Dynamic mechanical analysis, WCA, NMR, gel permeation chromatography (GPC)	[55]
	1%	84	11.7		
	3%	89	10.4		
	6%	86	13.8		
	10%	75	15.1		
Corn zein	(3 mg/cm²)	10.2 MPa-	3.3%-	SEM, release properties, antimicrobial activity, antioxidant capacity	[92]
	Catechin	1.1	142.2		
	Gallic acid	0.5	182.4		
	p-hydroxybenzoic acid	0.5	188.6		
	Ferulic acid	0.7	135.1		
	Flavone	6.7	2.21		
	Quercetin	5.3	1.23		
Corn zein	Lauryl chloride:zein	3.5 MPa-	46.8%-	NMR, FT-IR, SDS-PAGE, DSC, AFM	[94]
	20:1 mol%	2.8	101.9		
	60:1 mol%	3.0	163.1		
	100:1 mol%	3.7	302.3		
Soy protein and zein	soy protein Palmitoyl 1.6 mol/g Palmitoyl 4.8 mol/g zein Nonenyl-succinic anh. Dodecenyl-succinic anh. (each 2.5 mol/g)	(MPa) 2.65 0.44 1.31 1.84	[%] 33 50 0.35 1.6	DSC, TGA, FT-IR	[95]
